# Supplementary material for: Iterative improvement in the automatic modular design of robot swarms
Source: PeerJ Comput Sci. 2020 Dec 7;6:e322. doi: 10.7717/peerj-cs.322 (PMC7924708; doi:10.7717/peerj-cs.322)
Supplement: Supplemental Information 3 [file peerj-cs-06-322-s003.zip › argos3/doc/api/standalone/a00570_source.html]

ARGoS: plugins/simulator/entities/ground\_sensor\_equipped\_entity.cpp Source File


- Main Page
- Related Pages
- Namespaces
- Classes
- Files

- File List
- File Members

# plugins/simulator/entities/ground\_sensor\_equipped\_entity.cpp

Go to the documentation of this file.

```
00001 
00007 #include "ground_sensor_equipped_entity.h"
00008 #include <argos3/core/simulator/space/space.h>
00009 #include <argos3/core/simulator/entity/composable_entity.h>
00010 
00011 namespace argos {
00012 
00013    /****************************************/
00014    /****************************************/
00015 
00016    CGroundSensorEquippedEntity::CGroundSensorEquippedEntity(CComposableEntity* pc_parent) :
00017       CEntity(pc_parent) {
00018       Disable();
00019    }
00020    
00021    /****************************************/
00022    /****************************************/
00023 
00024    CGroundSensorEquippedEntity::CGroundSensorEquippedEntity(CComposableEntity* pc_parent,
00025                                                                   const std::string& str_id) :
00026       CEntity(pc_parent, str_id) {
00027       Disable();
00028    }
00029 
00030    /****************************************/
00031    /****************************************/
00032 
00033    CGroundSensorEquippedEntity::~CGroundSensorEquippedEntity() {
00034       while(! m_tSensors.empty()) {
00035          delete m_tSensors.back();
00036          m_tSensors.pop_back();
00037       }
00038    }
00039 
00040    /****************************************/
00041    /****************************************/
00042 
00043    void CGroundSensorEquippedEntity::Init(TConfigurationNode& t_tree) {
00044       try {
00045          /*
00046           * Parse basic entity stuff
00047           */
00048          CEntity::Init(t_tree);
00049          /*
00050           * Parse ground sensors
00051           */
00052          /* Not adding any sensor is a fatal error */
00053          if(t_tree.NoChildren()) {
00054             THROW_ARGOSEXCEPTION("No sensors defined");
00055          }
00056          /* Go through children */
00057          TConfigurationNodeIterator it;
00058          for(it = it.begin(&t_tree); it != it.end(); ++it) {
00059             std::string strAnchorId;
00060             GetNodeAttribute(*it, "anchor", strAnchorId);
00061             /*
00062              * NOTE: here we get a reference to the embodied entity
00063              * This line works under the assumption that:
00064              * 1. the GroundSensorEquippedEntity has a parent;
00065              * 2. the parent has a child whose id is "body"
00066              * 3. the "body" is an embodied entity
00067              * If any of the above is false, this line will bomb out.
00068              */
00069             CEmbodiedEntity& cBody = GetParent().GetComponent<CEmbodiedEntity>("body");
00070             if(it->Value() == "sensor") {
00071                CVector2 cOffset;
00072                GetNodeAttribute(*it, "offset", cOffset);
00073                std::string strType;
00074                GetNodeAttribute(*it, "type", strType);
00075                AddSensor(cOffset, ParseType(strType), cBody.GetAnchor(strAnchorId));
00076             }
00077             else if(it->Value() == "ring") {
00078                CVector2 cRingCenter;
00079                GetNodeAttributeOrDefault(t_tree, "center", cRingCenter, cRingCenter);
00080                Real fRadius;
00081                GetNodeAttribute(t_tree, "radius", fRadius);
00082                CDegrees cRingStartAngleDegrees;
00083                GetNodeAttributeOrDefault(t_tree, "start_angle", cRingStartAngleDegrees, cRingStartAngleDegrees);
00084                CRadians cRingStartAngleRadians = ToRadians(cRingStartAngleDegrees);
00085                std::string strType;
00086                GetNodeAttribute(*it, "type", strType);
00087                ESensorType eType = ParseType(strType);
00088                UInt32 unNumSensors;
00089                GetNodeAttribute(t_tree, "num_sensors", unNumSensors);
00090                AddSensorRing(cRingCenter,
00091                              fRadius,
00092                              cRingStartAngleRadians,
00093                              eType,
00094                              unNumSensors,
00095                              cBody.GetAnchor(strAnchorId));
00096             }
00097             else {
00098                THROW_ARGOSEXCEPTION("Unrecognized tag \"" << it->Value() << "\"");
00099             }
00100          }
00101       }
00102       catch(CARGoSException& ex) {
00103          THROW_ARGOSEXCEPTION_NESTED("Initialization error in ground sensor equipped entity", ex);
00104       }
00105    }
00106 
00107    /****************************************/
00108    /****************************************/
00109 
00110    void CGroundSensorEquippedEntity::Enable() {
00111       CEntity::Enable();
00112       for(size_t i = 0; i < m_tSensors.size(); ++i) {
00113          m_tSensors[i]->Anchor.Enable();
00114       }
00115    }
00116 
00117    /****************************************/
00118    /****************************************/
00119 
00120    void CGroundSensorEquippedEntity::Disable() {
00121       CEntity::Disable();
00122       for(size_t i = 0; i < m_tSensors.size(); ++i) {
00123          m_tSensors[i]->Anchor.Disable();
00124       }
00125    }
00126 
00127    /****************************************/
00128    /****************************************/
00129 
00130    void CGroundSensorEquippedEntity::AddSensor(const CVector2& c_offset,
00131                                                ESensorType e_type,
00132                                                SAnchor& s_anchor) {
00133       m_tSensors.push_back(new SSensor(c_offset, e_type, s_anchor));
00134    }
00135 
00136    /****************************************/
00137    /****************************************/
00138    
00139    void CGroundSensorEquippedEntity::AddSensorRing(const CVector2& c_center,
00140                                                       Real f_radius,
00141                                                       const CRadians& c_start_angle,
00142                                                       ESensorType e_type,
00143                                                       UInt32 un_num_sensors,
00144                                                       SAnchor& s_anchor) {
00145       CRadians cSensorSpacing = CRadians::TWO_PI / un_num_sensors;
00146       CRadians cAngle;
00147       CVector2 cOffset;
00148       for(UInt32 i = 0; i < un_num_sensors; ++i) {
00149          cAngle = c_start_angle + i * cSensorSpacing;
00150          cAngle.SignedNormalize();
00151          cOffset.Set(f_radius, 0.0f);
00152          cOffset.Rotate(cAngle);
00153          cOffset += c_center;
00154          AddSensor(cOffset, e_type, s_anchor);
00155       }
00156    }
00157 
00158    /****************************************/
00159    /****************************************/
00160 
00161    CGroundSensorEquippedEntity::ESensorType CGroundSensorEquippedEntity::ParseType(const std::string& str_type) const {
00162       if(str_type == "bw")    return TYPE_BLACK_WHITE;
00163       if(str_type == "gray") return TYPE_GRAYSCALE;
00164       THROW_ARGOSEXCEPTION("Unrecognized ground sensor type \"" << str_type << "\"");
00165    }
00166 
00167    /****************************************/
00168    /****************************************/
00169 
00170    REGISTER_STANDARD_SPACE_OPERATIONS_ON_ENTITY(CGroundSensorEquippedEntity);
00171    
00172    /****************************************/
00173    /****************************************/
00174 
00175 }
```

---

Generated on 10 Jul 2018 for ARGoS by 
 1.6.1
